# Long-lasting BDNF signaling alterations in the amygdala of adolescent female rats exposed to the activity-based anorexia model

**DOI:** 10.3389/fnbeh.2022.1087075

**Published:** 2022-12-08

**Authors:** Francesca Mottarlini, Beatrice Rizzi, Giorgia Targa, Fabio Fumagalli, Lucia Caffino

**Affiliations:** Laboratory of Experimental Psychopharmacology, Department of Pharmacological and Biomolecular Sciences, Università degli Studi di Milano, Milano, Italy

**Keywords:** activity-based anorexia, adolescence, amygdala, BDNF, caloric restriction, physical activity

## Abstract

**Introduction:** Anorexia nervosa (AN) is a severe psychiatric disorder characterized by a pathological fear of gaining weight, excessive physical exercise, and emotional instability. Since the amygdala is a key region for emotion processing and BDNF has been shown to play a critical role in this process, we hypothesized that alteration in the amygdalar BDNF system might underline vulnerability traits typical of AN patients.

**Methods:** To this end, adolescent female rats have been exposed to the Activity-Based Anorexia (ABA) protocol, characterized by the combination of caloric restriction and intense physical exercise.

**Results:** The induction of the anorexic phenotype caused hyperactivity and body weight loss in ABA animals. These changes were paralleled by amygdalar hyperactivation, as measured by the up-regulation of *cfos* mRNA levels. In the acute phase of the pathology, we observed reduced *Bdnf exon IX, exon IV*, and *exon VI* gene expression, while mBDNF protein levels were enhanced, an increase that was, instead, uncoupled from its downstream signaling as the phosphorylation of TrkB, Akt, and S6 in ABA rats were reduced. Despite the body weight recovery observed 7 days later, the BDNF-mediated signaling was still downregulated at this time point.

**Discussion:** Our findings indicate that the BDNF system is downregulated in the amygdala of adolescent female rats under these experimental conditions, which mimic the anorexic phenotype in humans, pointing to such dysregulation as a potential contributor to the altered emotional processing observed in AN patients. In addition, since the modulation of BDNF levels is observed in other psychiatric conditions, the persistent AN-induced changes of the BDNF system in the amygdala might contribute to explaining the onset of comorbid psychiatric disorders that persist in patients even beyond recovery from AN.

## Introduction

Anorexia nervosa (AN) is a severe psychiatric disease, which apart from the most known symptoms as restricted diet, strenuous exercise regimens, and severe emaciation (DSM-V), it is considered to be an ego syntonic disease, highlighting the impact of emotional domains in the pathophysiology of AN (O’hara et al., 2015). Many individuals consciously deny the seriousness of their undernourished state, indeed AN still shows the highest mortality and relapse rate among psychiatric disorders (Arcelus et al., 2011). A core component of the disease is indeed represented by altered emotional processing, that strongly influences the strict control of food intake in AN patients who show anxious and obsessional traits (Lavender et al., 2015; Kucharska et al., 2019). In particular, deficits in the flexible use of emotion regulation strategies and in emotional reactivity to disorder relevant stimuli, such as food and body images, are hallmarks of AN (Zhu et al., 2012). Moreover, maladaptive emotional processing has been found in longitudinal studies to predict the severity of AN symptoms (Racine and Wildes, 2015; Oldershaw et al., 2018). Despite the clear symptomatology, an in-depth investigation of the neurobiological underpinnings of emotion regulation impairments in AN is still lacking.

Imaging studies with functional magnetic resonance in individuals with AN revealed reduced functional connectivity in the fronto-amigdalar circuit, thus suggesting that structural and functional dysregulation of these brain areas are likely to underpin deficits in emotional regulation in AN (Steward et al., 2022) in line with other psychiatric disorders, such as depression, anxiety and post-traumatic stress disorders (Satterthwaite et al., 2016). Akin to this abnormal connectivity, disrupted maturation and altered activation of key regions required to successfully employ adaptive strategies in emotion processing, such as the amygdala (Amy; Steward et al., 2018), might drive a maladaptive shift in emotion regulation also in AN. Interestingly, Amy is reduced in volume and hyperactivated in AN patients (Burkert et al., 2019) and this hyperactivation is associated with body-image fearful emotional thoughts (Gaudio and Quattrocchi, 2012; Pruis et al., 2012; Simon et al., 2019). In particular, adolescent anorexic patients displayed elevated Amy reactivity when exposed to aversive pictures in comparison to a healthy control group (Seidel et al., 2018). Moreover, functional alterations of Amy compromise the processing of emotional stimuli in AN patients even after recovery (Phillips et al., 2003; Bang et al., 2016), suggesting the involvement of this brain region in the maintenance of the anorexic phenotype and in long-term AN-induced vulnerability.

From a neurobiological point of view, amygdalar-dependent responses to emotional stimuli were found to be mediated by the neurotrophin Brain-derived Neurotrophic factor (BDNF), a key molecule critically involved in neuroplastic mechanisms, memory processes and in the development of different psychiatric illnesses (Caffino et al., 2019b; Lorenzetti et al., 2020; Mottarlini et al., 2020b; Wang et al., 2022). Recently, BDNF has been involved in the pathophysiology of AN since it also regulates energy homeostasis *via* modulation of exigenic and anorexigenic pathways in the hypothalamus (Rosas-Vargas et al., 2011) and modifications in the expression and function of BDNF have been implicated in the alteration of food intake and body weight (Nakazato et al., 2012; Monteleone and Maj, 2013). According to genetic studies, Met/Met polymorphism of the BDNF gene has been associated with minimum body mass index in healthy adults, more severe disorder-related symptoms as well as risk of developing AN in both humans and mice (Ribases et al., 2003; Notaras et al., 2015; Clarke et al., 2016; Chen et al., 2017). In addition, several lines of evidence have shown that BDNF serum levels are significantly diminished in patients with AN and positively correlated with body mass index (Nakazato et al., 2003; Monteleone et al., 2004; Shobeiri et al., 2022). Notably, BDNF has been proposed as a target of food restriction and physical activity within mesocorticolimbic structures in the well-established experimental model of AN, the activity-based anorexia (ABA) model (Ho et al., 2016), pointing to its modulation as a signal of altered processing of food reward in both humans and experimental models.

Based on these lines of evidence, we hypothesized that the induction of the anorexic phenotype would dysregulate the BDNF system in the Amy, thus contributing to explaining the onset of comorbid psychiatric symptoms that might underline vulnerability traits typical of AN patients (Pollice et al., 1997). To this end, we exposed female adolescent rats to the ABA protocol, which consists of a combination of food restriction and physical exercise on a wheel, to mimic the hallmarks of AN, from the post-natal day (PND) 38 to PND42. Since the reduced activity of the BDNF-mediated signaling in Amy might be positively correlated with altered emotional regulation that, in turn, may facilitate relapse, we evaluated BDNF gene and protein expression and its signaling pathway across the induction and after a recovery phases from the ABA paradigm. In particular, since the *Bdnf* gene has a complex structure that consists of eight 5’ untranslated exons and one 3’ exon coding for the protein (Aid et al., 2007), *Bdnf exon IX*, representing the entire pool of transcripts, *exon IV*, the activity-dependent exon of somatic origin, and *exon VI* (Baj et al., 2011), the isoform known to be targeted to dendrites (Chiaruttini et al., 2008), mRNA levels have been evaluated. Moreover, together with these multiple transcripts the subcellular distribution of the mature form of the BDNF protein (mBDNF) together with the activation of the BDNF-mediated signaling pathway have been investigated. In line with our previously published manuscripts (Mottarlini et al., 2020a; 2022), the ABA-induced alterations were performed at the achievement of the anorexic phenotype (acute phase, represented by the 25% of weight loss and wheel activity increasing over days) and after a 7-day period of body weight recovery to identify possible molecular scars that might persist in the brain even when the body weight is restored.

## Materials and Methods

### Animals and housing

Adolescent female Sprague-Dawley rats were purchased from Charles River (Calco, Italy). Upon their arrival animals were housed in groups of four rats per cage, with a reversed 12 h light/dark cycle (light on/off: 10.30 p.m./10.30 a.m.), under standard conditions of temperature (21 ± 1°C) and humidity (50%–60%). They were fed with standard rat chow (ssniff Spezialdiäten GmbH, Soest, Germany) and tap water *ad libitum*. A maximum of two female siblings was taken from each litter to reduce the “litter effect” (Chapman and Stern, 1978).

All the procedures were conducted at the Department of Pharmacological and Biomolecular Science (University of Milan, Italy) and performed according to the principles set out in the following laws and policies governing the care and use of laboratory animals: Italian Governing Law (D.lgs 26/2014; Authorization n.19/2008-A issued March 6, 2008, by Ministry of Health); the NIH Guide for the Care and Use of Laboratory Animals (2011 edition) and EU directives and guidelines (EEC Council Directive 2010/63/UE). All efforts were made to minimize animal suffering and to employ the lowest number of animals: for ethical reasons, ABA rats were not allowed to lose more than 25% of their initial body weight. The experiments have been reported in compliance with the ARRIVE guidelines.

### Experimental design

**Figure 1:**
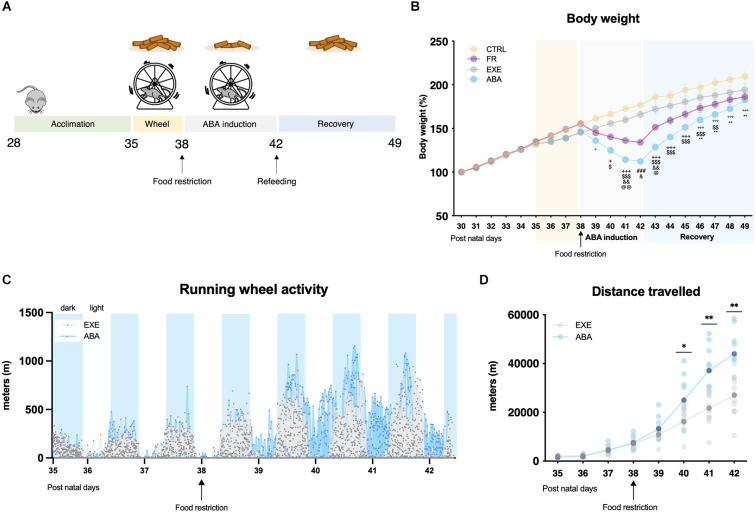
Graphical representation of the experimental paradigm for the induction of anorexia nervosa (AN) in adolescent female rats by means of the ABA protocol **(A)**. Average daily body weight **(B)** measured in control (CTRL), food-restricted (FR), exercise (EXE), and ABA rats. Total distance traveled over days were calculated in 30 min intervals **(C)** and averaged total distance traveled **(D)** for EXE and ABA rats. Results are presented as the mean ± SEM of five rats per group. Two-way ANOVA, followed by Bonferroni’s multiple comparisons test. °*p* < 0.05, ^°°°^*p* < 0.001 ABA vs. CTRL; ^+^*p* < 0.05, ^+++^*p* < 0.001 FR and ABA vs. CTRL; ^$^*p* < 0.05, ^$$^*p* < 0.01, ^$$$^*p* < 0.001 ABA vs. EXE; and ^&^*p* < 0.05, ^&&^*p* < 0.01 FR vs. ABA; ^@^*p* < 0.05, ^@@^*p* < 0.01 FR vs. EXE; ^###^*p* < 0.001 FR and ABA vs. CTRL and EXE; *p < 0.05, **p < 0.01 CTRL vs. FR. CTRL, control; FR, food-restricted; EXE, exercise; ABA, activity-based anorexia.

At their arrival (PND28), rats were group-housed, left undisturbed in their cages to habituate to the light cycle for 7 days and fed with food and water *ad libitum*. At PND35, animals were individually housed in transparent cages and randomly subdivided into four experimental groups: (1) control (CTRL, *n* = 10) group: sedentary + food *ad libitum*; (2) food-restricted (FR, *n* = 10) group: sedentary + food restriction (food access limited for 2 h/day); (3) exercise (EXE, *n* = 10) group: voluntary running activity in a mechanical wheel + food *ad libitum*; and (4) activity-based anorexia (ABA, *n* = 10) group: voluntary running activity in a mechanical wheel + food restriction (food access limited for 2 h/day). At the beginning of the experimental paradigm, as shown in Figure 1A, all animals started a 3-day acclimatization period (PND35-PND38) to the new housing condition, in which CTRL and FR groups were kept in classical home cages, while EXE and ABA groups were placed in dedicated activity cages equipped with a mechanical activity wheel (activity wheel BIO-ACTIVW-R cage, Bioseb, France). The habituation period was scheduled to reduce potential different individual aptitudes to exercise activity, to set the running baseline and to accustom animals to the interaction with the investigator. At PND38 the food restriction period started: FR and ABA rodents had access to the food pellets for 2 h/day from 10.30 to 12.30 a.m., a period during which the wheel was blocked to preclude rats from privileging run-over food.

At PND42, half of the animals per group (*n* = 5/group) were sacrificed by decapitation as they reached the acute phase of the anorexic phenotype, while the remaining animals (*n* = 5/group) were placed back in classical home cages and preserved as sedentary with food *ad libitum* for 7 days until PND49 to allow body weight recovery. After decapitation, amygdala tissues were grossly dissected from 2 mm slice following the coordinates (−1.72 mm to −3.24 mm bregma) of the Rat Brain Atlas of Paxinos and Watson (2013), frozen on dry ice, and stored at −80°C. Trunk blood from each rat was collected immediately after decapitation as well.

### Measurements

Body weight and food intake were evaluated per each animal from the first day of acclimatization (PND35) until the end of the experiment (PND42 or PND49). Rats were weighed daily between 8.00 and 9.00 a.m., before the dark shift. Food intake was measured with a specific milligram-sensitive scale before the beginning of the 2 h of food restriction and immediately after and calculated as grams of food given at the beginning of the 2 h of food access—grams weighted after the 2 h (see Supplementary Materials).

Running wheel activity was assessed by means of specific activity cages equipped with mechanical wheels (Activity wheel BIO-ACTIVW-R cage, Bioseb, Vitrolles, France) connected to a counter device associated with a monitoring software (BIO-ACTIVW-SOFT v1.2.1, Bioseb, Vitrolles, France). Running wheel data were recorded at 30 min intervals throughout the whole experiment and the distance traveled (m) is presented as average for each animal during the 24 h of recording (Figure 1C) and as individual determinations and as average during each experimental day (Figure 1D).

### mRNA extraction and real-time PCR analysis

Total RNA from amygdala tissues was isolated by single step guanidinium isothiocyanate/phenol extraction using PureZol RNA isolation reagent (Bio-Rad Laboratories, Italy) according to the manufacturer’s instructions, quantified by means of the Nanodrop spectrophotometric analysis and stored at −20°C until further processing.

Following total RNA extraction, the samples were processed for real-time reverse transcription polymerase chain reaction (real-time RT-PCR) to assess mRNA levels, as previously described (Caffino et al., 2019a). Briefly, an aliquot of each sample was treated with DNase (DNase I, RNase-free buffer di MnCl2-Thermo Scientific) to remove any genomic DNA still present, for 30 min at 37°C and, afterwards, incubated at 65°C for 10 min with EDTA to block DNase action. Expression levels of the genes of interest were analyzed by TaqMan qRT-PCR 48 thermal cycler (CFX384 real-time system, Bio-Rad Laboratories) using the iScriptTM one-step RT-PCR kit for probes (Bio-Rad Laboratories). Samples were run in triplicate in a 384-wells plate. Thermal cycling was initiated with incubation at 50°C for 10 min (RNA retrotranscription) and then at 95°C for 5 min (TaqMan polymerase activation). After this initial step, 39 cycles of PCR were performed. Each PCR cycle consisted of heating the samples at 95°C for 10 s to enable the melting process and then for 30 s at 60°C for the annealing and extension reaction. Data were analyzed with the comparative threshold cycle (ΔΔCt) using the geometric means of *36B4, β-actin*, and *18S* gene as internal standard (Caffino et al., 2015).

RT-PCR analysis was performed to evaluate *Bdnf exon IX* gene expression as well as the expression of *Bdnf exons IV* and *VI* and *cfos*. Primers and probe for *Bdnf exon IV* and *VI* were purchased from Applied Biosystem, Foster City, California (*Bdnf exon IV*: ID Rn01484927_m1 and *Bdnf exon VI*: ID Rn01484928_m1). Primers and probes for *Bdnf exon IX, cfos, 36B4, β-Actin, and 18S* were purchased from Eurofins MWG-Operon. Their sequences are shown below:

- *Bdnf exon IX*: forward primer 5’-AAGTCTGCATTACATTCCTCGA-3’, reverse primer 5’-GTTTTCTGAAAGAGGGACAGTTTAT-3’, probe 5’- TGTGGTTTGTTGCCGTTGCCAAG-3’;
- *cfos*: forward primer 5’-TCCTTACGGACTCCCCAC-3’, reverse primer 5’-CTCCGTTTCTCTTCCTCTTCAG-3’, probe 5’-TGCTCTACTTTGCCCCTTCTGCC-3’;
- *36B4*: forward primer 5’-TTCCCACTGGCTGAAAAGGT-3’, reverse primer 5’-CGCAGCCGCAAATGC-3’, probe 5’-AAGGCCTTCCTGGCC GATCCATC-3’;
- β-Actin: forward primer 5’- CACTTTCTACAATGAGCTGCG-3’, reverse primer 5’- CTGGATGGCTACGTACATGG-3’, probe 5’-TCTGGGTCATCTTTTCACGGTTGGC-3’;
- *18S*: forward primer 5’- GTAACCCGTTGAACCCCATT-3’, reverse primer 5’- CCATCCAATCGGTAGTAGCG-3’, probe 5’- TGCAATTATTCCCCATGAACGAGG-3’.

### Protein extracts preparation and Western blot analysis

Proteins from Amy tissues were homogenized using a cold buffer containing 0.32 M sucrose, 0.1 mM PMSF, 1 mM HEPES, 0.1 mM EGTA pH 7.4, in presence of commercial cocktails of protease (Roche-Merk Life Science, Milan, Italy), and phosphatase (Sigma-Aldrich-Merk Life Science, Milan, Italy) inhibitors and crude membrane fraction prepared as previously described (Caffino et al., 2018). The total amount of proteins in the crude membrane fraction and in the homogenate was quantified according to the Bradford Protein Assay procedure (Bio-Rad, Milan, Italy), with bovine serum albumin as the calibration standard. Samples were stored at −20°C until molecular analysis.

Eight micrograms of proteins for each sample were run on a sodium dodecyl sulfate—14% polyacrylamide gel under reducing conditions and electrophoretically transferred onto a nitrocellulose membrane (Bio-Rad Laboratories). Blots were blocked for 1 h at room temperature with I-Block solution (Life Technologies Italia) in TBS + 0.1% Tween-20 buffer, incubated with antibodies against the phosphorylated forms of the proteins, and then stripped and reprobed with the antibodies against corresponding total proteins.

Results were standardized to the β-actin control protein detected at 43 kDa. Immunocomplexes were visualized by chemiluminescence using the Chemidoc MP Imaging System (Bio-Rad Laboratories) and analyzed with Image LabTM software (Bio-Rad) by evaluating the band density (Figures 5L, 6G). As gels were run in duplicate, the results from the two gels were averaged with a correction factor: correction factor gel B = average of (OD protein of interest/OD β-actin for each sample loaded in gel A)/(OD protein of interest/OD β-actin for the same sample loaded in gel B; Caffino et al., 2020).

**Figure 2:**
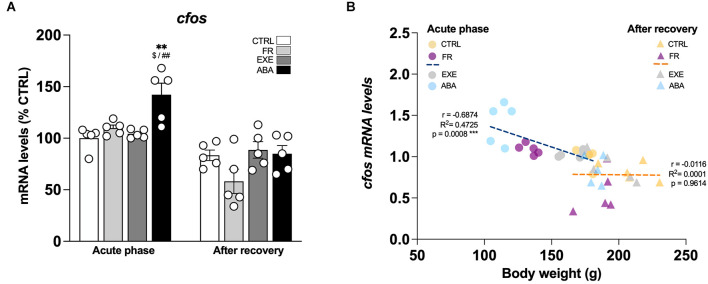
Effects of the ABA protocol on *cfos* gene expression levels in the amygdala. Rats were exposed to the combination of food restriction and free access to the activity wheel and the analysis of *cfos* mRNA levels in the amygdala **(A)** was performed in the acute phase of the disease (PND42) and after a 7-day period of body weight recovery (PND49). Data are expressed as percentages of control (CTRL) animals sacrificed in the acute phase. Bar graphs represent the mean ± SEM from five independent determinations for each experimental group. Pearson’s product–moment correlation (r) analyses and linear regression analyses (R^2^) between body weight and *cfos* **(B)** mRNA levels of CTRL, FR, EXE, and ABA rats are represented. Two-way ANOVA followed by Tukey’s multiple comparisons test. **p < 0.01 vs. CTRL-acute phase; ^$^*p* < 0.05 vs. FR-acute phase; ^##^*p* < 0.01 vs. EXE-acute phase. CTRL, control; FR, food-restricted; EXE, exercise; ABA, activity-based anorexia.

**Figure 3:**
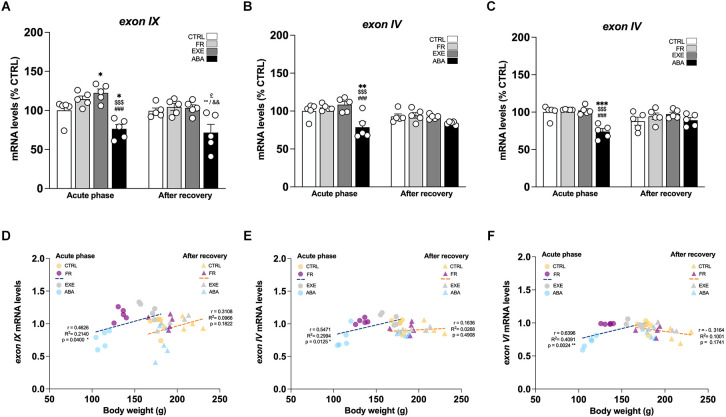
Effects of the ABA protocol on *Bdnf* gene expression levels in the amygdala. Rats were exposed to the combination of food restriction and free access to the activity wheel and the analysis of *Bdnf exon IX* **(A)**, *exon IV* **(B)**, and *exon VI* **(C)** mRNA levels in the amygdala was performed in the acute phase of the disease (PND42) and after a 7-day period of body weight recovery (PND49). Data are expressed as percentages of control (CTRL) animals sacrificed in the acute phase. Bar graphs represent the mean ± SEM from five independent determinations for each experimental group. Pearson’s product–moment correlation (r) analyses and linear regression analyses (R^2^) between body weight and *total Bdnf* **(D)**, *exon IV* **(E)**, and *exon VI* **(F)** mRNA levels of CTRL, FR, EXE, and ABA rats are represented. Three-way ANOVA or two-way ANOVA followed by Tukey’s multiple comparisons test. *p < 0.05, **p < 0.01, ***p < 0.05 vs. CTRL-acute phase; ^$$$^*p* < 0.001 vs. FR-acute phase; ^###^*p* < 0.001 vs. EXE-acute phase; ^£^*p* < 0.05 vs. CTRL-recovery; ^°°^*p* < 0.01 vs. FR-recovery; ^&&^*p* < 0.01 vs. EXE-recovery. CTRL, control; FR, food-restricted; EXE, exercise; ABA, activity-based anorexia.

**Figure 4:**
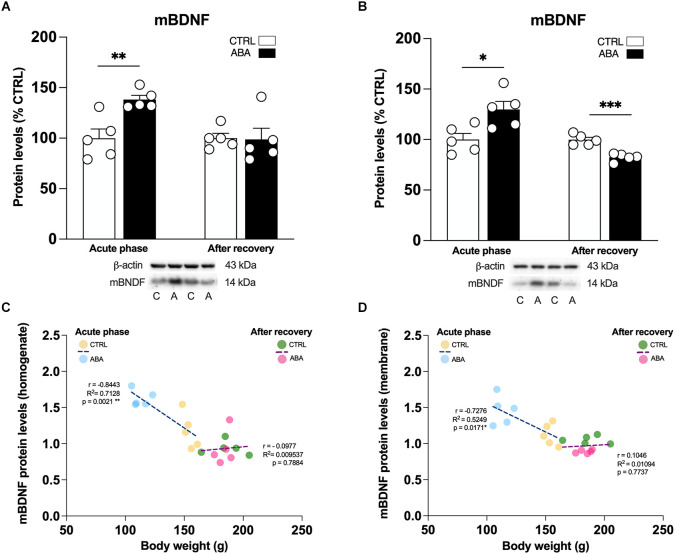
Effects of the ABA protocol on mBDNF protein levels in the amygdala. Rats were exposed to the combination of food restriction and free access to the activity wheel and the analysis of mBDNF in the whole homogenate **(A)**, and in the crude membrane fraction **(B)** were performed in the acute phase of the disease (PND42), and after a 7-day period of body weight recovery (PND49). Pearson’s product–moment correlation (r) analyses and linear regression analyses (R^2^) between body weight and mBDNF in the homogenate **(C)** and in the crude membrane fraction **(D)** are represented. Below the graphs representative immunoblots are shown for mBDNF (14 kDa) and β-Actin (43 kDa) proteins. Data are expressed as percentages of control (CTRL) animals. Bar graphs represent the mean ± SEM from five independent determinations for each experimental group. Unpaired Student’s *T* test. *p < 0.05, **p < 0.01, ***p < 0.05. CTRL or C, control; ABA or A, activity-based anorexia.

**Figure 5:**
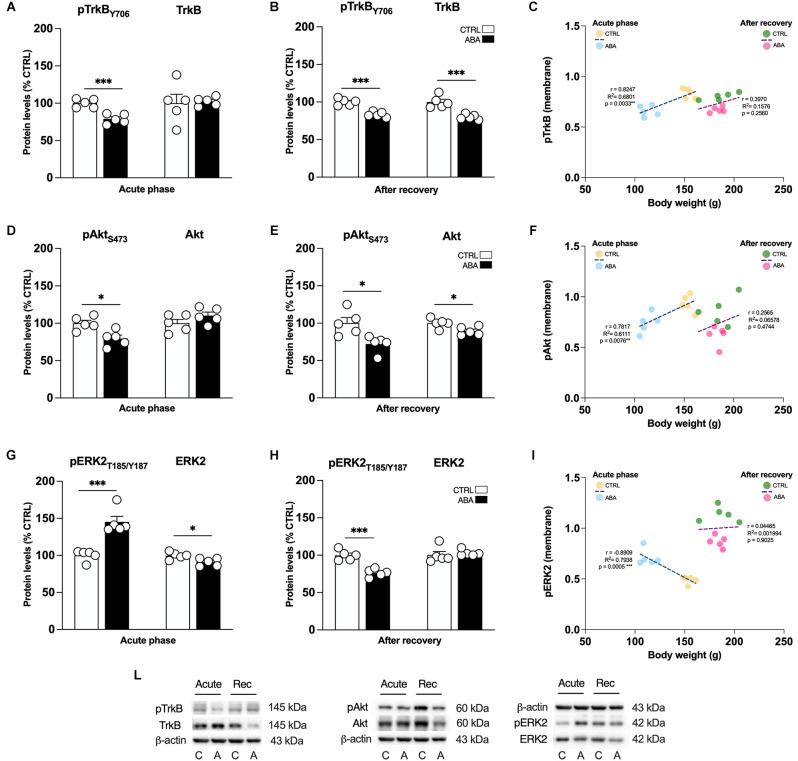
Effects of the ABA protocol on the protein levels of TrkB, the high affinity receptor for mBDNF, and on its downstream signaling effectors in the amygdala. Rats were exposed to the combination of food restriction and free access to the activity wheel and the analysis of phospho(p)TrkBY706 **(A)**, TrkB receptor **(B)**, pAktS473 **(D)**, Akt **(E)**, pERK2T185-Y187 **(G)**, and ERK2 **(H)** were measured in the crude membrane fraction in the acute phase of the disease (PND42), and after a 7-day period of body weight recovery (PND49). Pearson’s product–moment correlation (r) analyses and linear regression analyses (R^2^) between body weight and pTrkBY706 **(C)**, pAktS473 **(F)**, and pERK2T185-Y187 **(I)** phosphorylation levels of CTRL and ABA rats at both time points are represented. In panel **(L)**, representative immunoblots are shown for pTrkB Y706 (145 kDa), TrkB (145 kDa), pAktS473, Akt, pERK2T185-Y187, ERK2, and β-Actin (43 kDa). Data are expressed as percentages of CTRL animals. Bar graphs represent the mean ± SEM from five independent determinations for each experimental group. Unpaired Student’s *T* test. *p < 0.05, **p < 0.01, ***p < 0.05. CTRL or C, control; ABA or A, activity-based anorexia.

The conditions of the primary antibodies were the following: anti-mBDNF (1:500, Icosagen, cod. 327-100); anti-pTrkB tyr706 (1:200, Novus Biologicals, cod. NBP2-54764); anti-TrkB (1:1,000 Cell Signaling Technology cod. 4606, RRID:AB_2267470); anti-pAkt ser473 (1:1,000, Cell Signaling Technology cod. 4060, RRID:AB_2315049); anti-Akt (1:1,000, Cell Signaling Technology, cod. 9272, RRID:AB_329827); anti-pERK2 thr185/tyr187 (1:1,000, Cell Signaling Technology cod. 4370, RRID:AB_2315112); anti-ERK2 (1:2,000, Cell Signaling Technology cod. 4695, RRID:AB_390779); anti-p-mTOR ser2448 (1:1,000 Cell Signaling Technology Inc., Antibody cod. 2971, RRID:AB_330970), anti-mTOR (1:1,000 Cell Signaling Technology Inc., Antibody cod. 2972, RRID:AB_330978), anti-p-S6 ser240/244 (1:1,000 Cell Signaling Technology Inc., Antibody cod. 9468, RRID:AB_2716873), anti-S6 (1:1,000 Cell Signaling Technology Inc., Antibody cod. 2217, RRID:AB_331355), and anti-β-actin (1:5,000, Sigma-Aldrich, cod. A5441, RRID:AB_476744).

### Plasma collection and BDNF ELISA assay

Trunk blood from each rat was collected immediately after decapitation in vials containing EDTA (0.5 M, pH 8). The total amount of blood was centrifuged at 3,000 *g* for 20 min to precipitate and remove the cellular fraction. The supernatant, corresponding to the plasma fraction, was stored at −80°C until the analysis. BDNF levels were determined by an enzyme-linked immunosorbent assay (ELISA) using commercial kits, according to the manufacturer’s instructions. The calibration curve was run in duplicate together with the control and samples, using the same procedure. BDNF concentration of each sample was calculated related to the standard calibration curve.

### Statistical analysis

Data were collected in individual animals as independent determinations and are reported as means and standard errors.

Behavioral data related to the characterization of the anorexic phenotype, such as body weight, distance traveled on the wheel and food intake were assessed by two-way analysis of variance (ANOVA) with repeated measures, followed by Bonferroni’s multiple comparisons test.

Molecular changes in mRNA levels were analyzed initially by a three-way (analysis of variance) ANOVA to investigate manipulations- and time-related differences, incorporating the following variables: food availability (food *ad libitum* vs. food restriction), physical activity (sedentary vs. exercise) and time of sacrifice (PND42 acute phase vs. PND49 after recovery). As dictated by the relevant interaction terms, low-order ANOVAs were used to determine manipulation effects and interactions followed by Tukey’s multiple comparisons test to characterize differences between groups. Detailed statistics, such as F and p values of independent variables of two-way or three-way ANOVA, were reported in the Supplementary Tables 1–5.

Molecular changes in protein levels were tested for normality of residuals with the Kolmogorov-Smirnov test. Data with normal distribution were analyzed by unpaired Student’s *t*-test (t), using as control condition the combination of food *ad libitum* and no physical exercise (CTRL), and as the testing condition, the combination of food restriction and physical exercise (ABA), which were considered as independent variables at the two different time points analyzed, PND42 or PND49, respectively. Data with a non-normal distribution were analyzed by the Mann-Whitney test (U).

Pearson’s product-moment coefficients (r) and linear regression analyses (R^2^) were calculated to study potential correlations between behavioral outcomes induced by the ABA procedure with molecular changes observed in the amygdala of ABA rodents.

Subjects were eliminated from the final dataset if their data deviated from the mean by 2 SDs. Prism 8 (GraphPad Software Prism v8, San Diego, CA, USA) was used for the analysis of all data. Significance for all tests was assumed at *p* < 0,05.

## Results

The induction of the anorexic phenotype provokes a substantial reduction of body weight, and it causes hyperactive behaviors only in female rats exposed to the combination of a restricted feeding schedule and wheel access, i.e., the ABA group. Consistent with our previous findings (Mottarlini et al., 2020a; 2022), after 24 h from the first day of food restriction (PND 39, Figure 1A), ABA rats reduced their body weight in respect to CTRL and EXE animals (Figure 1B: repeated measure two-way ANOVA manipulation *F*_(3,16)_ = 3.953, *p* = 0.0276; time *F*_(19,304)_ = 623.8, *p* < 0.0001; interaction *F*_(57,304)_ = 21.31, *p* < 0.0001), which were not subjected to food limitation. Such reduction progresses over days and, at PND 42, ABA rats reached the maximum allowed body weight loss, which was significantly greater even with respect to FR rats, despite the same quantity of food eaten (Supplementary Figure 1, Table 1). In parallel, ABA rats exhibit a strong increase of wheel running activity vs. EXE animals, highlighting an escalation of activity also during resting periods (Figure 1C) and an increase in the total distance traveled on the wheel over days (Figure 1D: repeated measure two-way ANOVA manipulation *F*_(1,18)_ = 7.685, *p* = 0.0126; *F*_(7,114)_ = 159.4, time *p* < 0.0001, interaction *F*_(7,114)_ = 13.80, *p* < 0.0001). During the recovery period (PND 42-PND 49), in which food was available *ad libitum* and wheel removed, ABA rats begin to regain weight without, however, returning to the weight of CTRL rats at the end of the experiment (Figure 1B), suggesting ABA rats need more time to achieve full recovery (Supplementary Table 2).

**Table 1:** Pearson’s product moment coefficients (r), linear regression analyses (R^2^), and p values relative to the correlation between food intake measured in grams (g) and mRNA or protein levels in CTRL, FR, EXE, and ABA rats at both the achievement of the anorexic phenotype and following a 7-day recovery period.

**Food intake (g)**	**Acute phase**				**After recovery**			
	**R**	**R^2^**	***p*-value**		**r**	**R^2^**	***p*-value**	
cFos	−0.6922	0.4792	0.0265	*	0.2554	0.06524	0.4763	
Bdnfexon IX	0.7684	0.5904	0.0094	**	−0.5681	0.3227	0.0867	
Bdnf exon IV	0.7160	0.5127	0.0199	*	−0.4221	0.1782	0.2243	
Bdnf exon VI	0.8822	0.7783	0.0007	***	−0.07708	0.005941	0.8324	
mBDNF(homogenate)	−0.8164	0.6665	0.0040	**	−0.0005032	2.532e-007	0.9989	
mBDNF(membrane)	−0.7350	0.5402	0.0154	*	−0.6707	0.4499	0.0338	*
pTrkBtyr706	0.7905	0.6248	0.0065	**	−0.6484	0.4204	0.0426	*
TrkB	−0.03380	0.001143	0.9261		−0.5793	0.3355	0.0793	
pAktser473	0.7051	0.4971	0.0228	*	−0.5600	0.3136	0.0923	
Akt	−0.3568	0.1273	0.3116		−0.6363	0.4049	0.0479	*
pERK2 thr185/tyr187	−0.8675	0.7525	0.0011	**	−0.7038	0.4954	0.0231	*
ERK2	0.7258	0.5269	0.0175	*	0.1702	0.02898	0.6382	
pmTORser2448	−0.9138	0.8350	0.0002	***	0.2726	0.07430	0.4461	
mTOR	−0.6129	0.3756	0.0596		−0.6187	0.3828	0.0565	
pS6 ser240/244	0.8596	0.7389	0.0030	**	−0.6750	0.4556	0.0322	*
S6	−0.03189	0.001017	0.9303		−0.5658	0.3201	0.0882	

*p < 0.05, **p < 0.01, ***p < 0.001.

The subsequent molecular analysis has been performed in the Amy of CTRL, FR, EXE, and ABA rodents at two time points: PND 42, which corresponds to the acute phase of the ABA induction, and PND 49, after a period of body weight restoration.

From a molecular standpoint, we first investigated the neuronal activation of the Amy in our experimental condition evaluating the expression of the immediate early gene *cfos*, a well-established marker of neuronal activation that links gene expression to synaptic plasticity (Morgan and Curran, 1989; Kaczmarek, 1993). In the acute phase, at PND42, only ABA rodents show a marked increase of *cfos* expression (Figure 2A: two-way ANOVA interaction *F*_(1,16)_ = 4.777, *p* = 0.0441), an effect that is negatively correlated with body weight (Figure 2B) and food intake (Table 1) and that was abolished after a one-week period of body weight recovery (Figure 2A: two-way ANOVA interaction *F*_(1,16)_ = 1.569, *p* = 0.2283).

Next, we focused our attention on the evaluation of the BDNF system. At PND 42, wheel activity in EXE rats increases *Bdnf exon IX* levels whereas the induction of the AN phenotype in ABA rats causes a reduction of *Bdnf exon IX* mRNA levels (Figure 3A: two-way ANOVA interaction *F*_(1,16)_ = 31.56, *p* < 0.0001), an effect that was maintained also after recovery, at PND 49 (two-way ANOVA interaction *F*_(1,16)_ = 9.629, *p* = 0.0068). Similarly, we observed reduced levels of mRNA also for *Bdnf exon IV* (Figure 3B: three-way ANOVA interaction *F*_(1,32)_ = 5.282, *p* = 0.0282), and *exon VI* (Figure 3C: two-way ANOVA interaction *F*_(1,16)_ = 25.98, *p* < 0.0001), at PND 42; these effects are restored at PND 49 after the recovery period (Figure 3B *exon IV*; Figure 3C exon *VI*: two-way ANOVA interaction *F*_(1,16)_ = 3.272, *p* = 0.0893). Pearson’s correlation and linear regression analyses were run to determine the relationship between ABA induction and the herein investigated molecular targets. As shown in Figure 3; Tables s 1 and 2, we observed that the reduced levels of *Bdnf exon IX* and *exons IV* and *VI* in the acute phase positively correlate with their body weight and food intake and negatively correlate with wheel running activity measured at PND 42, revealing a strong interaction between reduced levels of body weight and food intake and increased levels of physical activity and reduced levels of *Bdnf* mRNA levels (Figures 3D–F; Tables s 1 and 2), effects that were totally abolished at PND 49 (Figures 3D–F; Tables s 1 and 2).

**Table 2:** Pearson’s product moment coefficients (r), linear regression analyses (R^2^), and p values relative to the correlation between wheel running activity measured in meters (m) and mRNA levels in EXE and ABA rats at both the achievement of the anorexic phenotype and following a 7-day recovery period.

**Running activity (m)**	**Acute phase**				**After recovery**			
	**r**	**R^2^**	***p*-value**		**r**	**R^2^**	***p*-value**	
cFos	0.1494	0.02232	0.6804		−0.05350	0.002862	0.8833	
Bdnf exon IX	0.6804	0.5280	0.0173	*	−0.5017	0.2517	0.1396	
Bdnf exon IV	−0.8660	0.7500	0.0012	**	−0.6950	0.4830	0.0257	*
Bdnf exon VI	−0.7406	0.5485	0.0143	*	−0.03434	0.001179	0.9250	

*p < 0.05, **p < 0.01.

To determine whether changes in transcription are paralleled by changes in translation, we examined the expression of BDNF, the high affinity receptor TrkB and its signaling pathway in the amygdalar whole homogenate and crude membrane fraction. These analyses allow us to dissect the effect of ABA induction on BDNF protein translation, which derives from measurements in the whole homogenate, and from the availability of the neurotrophin at synaptic sites, depending on the analyses in the membrane fraction. Since the gene expression analysis presented in Figures 2 and 3 shows that only the combination of food restriction and hyperactivity specifically alters *cfos* and *Bdnf* transcripts, we decided to focus our attention on ABA vs. CTRL group in the study of protein levels to investigate the potential molecular mechanisms underlying such effect. As shown in Figure 3; ABA rats display increased levels of mBDNF protein levels in the acute phase both in the whole homogenate (Figure 4A: +38% vs. CTRL, *t* = 3.752, *p* = 0.0056) and membrane fraction (Figure 4B: +30% vs. CTRL, *t* = 2.995, *p* = 0.0172). At PND 49, no effects were observed in mBDNF protein levels in the homogenate (Figure 4A: −1% vs. CTRL, *U* = 8, *p* = 0.4206), whereas in the membrane fraction they were reduced (Figure 4B: −18% vs. CTRL, *t* = 6.015, *p* = 0.0003). Interestingly, Pearson’s correlations reveal that increased levels of mBDNF protein expression either in the whole homogenate (Figure 4C) or in the membrane (Figure 4D) are negatively correlated with body weight and food intake (Table 1), indicating that increased levels of mBDNF correspond to reduced food intake and low body weight. After recovery, the same analyses did not reveal any significant correlation with body weight (Figures 4C,D) while a negative correlation between food intake and mBDNF levels in the membrane fraction. At variance from BDNF protein levels, the phosphorylation of its high-affinity receptor TrkB in Tyr(Y)706 measured in the membrane fraction of the Amy is significantly reduced in ABA rats at both time points (Figure 5A: −21% vs. CTRL, *t* = 5.396, *p* = 0.0006; Figure 5B: −10% vs. CTRL, *t* = 6.677, *p* = 0.0002). No changes in its total expression were found in the acute phase (Figure 5A: +2% vs. CTRL, *t* = 0.1823, *p* = 0.8599), while after the period of recovery the total expression of TrkB was reduced (Figure 5B: −11% vs. CTRL, *t* = 5.073, *p* = 0.0010).

Next, to investigate whether alterations in the BDNF-TrkB system might have an impact on the BDNF-TrkB downstream signaling pathway we measured the levels of expression and phosphorylation of Akt and ERK2 effectors. As shown in Figure 5, the levels of Akt phosphorylation in serine (S) 473 were reduced in ABA rats in the acute phase (Figure 5D: −21% vs. CTRL, *t* = 3.333, *p* = 0.0103), while no changes were observed measuring the total levels of Akt protein expression (Figure 5D: +10% vs. CTRL, *t* = 1.436, *p* = 0.1889). Of note, the reduction of pAkt was present as well after the period of body weight recovery (Figure 5E: −28% vs. CTRL, *t* = 3.077, *p* = 0.0152) and in addition, at the same time point, also the total level of protein expression of Akt were reduced (Figure 5E: −11% vs. CTRL, *t* = 2.660, *p* = 0.0288). Interestingly, and contrary to pAkt, the phosphorylation levels of ERK2 protein in Threonine (T) 185/Tyrosine (Y) 187 were significantly increased in the acute phase of ABA induction (Figure 5G: +45% vs. CTRL, *t* = 5.560, *p* = 0.0005), while total expression of ERK2 was reduced (Figure 5G: −9% vs. CTRL, *t* = 2.933, *p* = 0.0189). After recovery, we observed a different picture: pERK2 was reduced (Figure 5H: −34% vs. CTRL, *t* = 6.176, *p* = 0.0003), and no significant changes were found in its total protein expression (Figure 5H: +3% vs. CTRL, *U* = 5 *p* = 0.1508).

**Figure 6:**
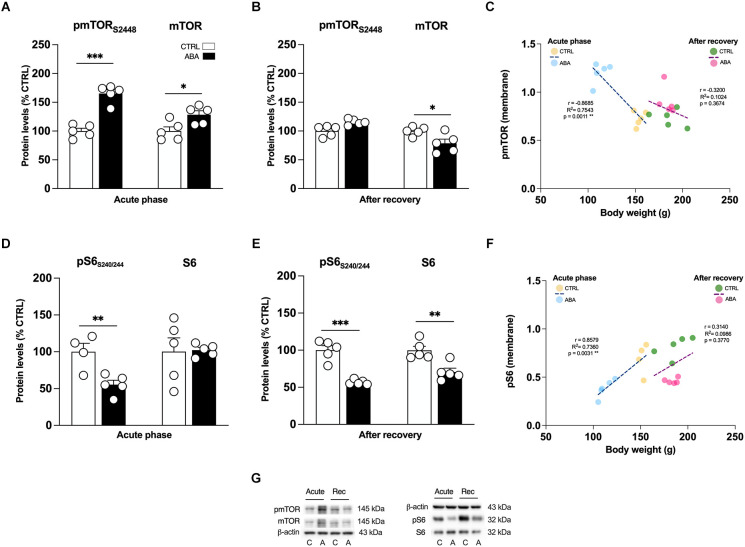
Effects of the ABA protocol on the protein levels of mTOR and on its downstream effector S6 in the amygdala. Rats were exposed to the combination of food restriction and free access to the activity wheel and the analysis of phospho(p)-mTOR S2448 **(A)**, and of mTOR **(B)**, pS6 S240/244 **(D)**, S6 **(E)** were measured in the crude membrane fraction at the acute phase of the disease (PND42), and after a 7-day period of body weight recovery (PND49). Pearson’s product–moment correlation (r) analyses and linear regression analyses (R^2^) between body weight and pmTOR S2448 **(C)**, pS6 S240/244 **(F)** phosphorylation levels of CTRL and ABA rats at both time points are represented. In panel **(G)**, representative immunoblots are shown for pmTOR S2448, mTOR, pS6 S240/244, S6, and β-Actin (43 kDa). Data are expressed as percentages of CTRL animals. Bar graphs represent the mean ± SEM from five independent determinations for each experimental group. Unpaired Student’s *T* test. *p < 0.05, **p < 0.01, ***p < 0.05. CTRL or C, control; ABA or A, activity-based anorexia.

Akt and ERK2 are well-known upstream regulators of mTOR, a key protein that regulates the initiation of protein translation, integrating both intracellular and extracellular signals fundamental in the control of protein synthesis required for the formation of new synaptic connections. In our model, we observed that the induction of the ABA protocol increased mTOR phosphorylation in serine (S) 2,448 and its total expression (Figure 6A: +63% vs. CTRL, *t* = 7.956, *p* < 0.0001; +14% vs. CTRL, *t* = 7.956, *p* < 0.0001). Conversely, after 7 days of body weight recovery, we observed no changes in mTOR phosphorylation and a significant reduction in mTOR expression (Figure 6B: +8% vs. CTRL, *U* = 5, *p* < 0.1508; −21% vs. CTRL, *t* = 2.678, *p* = 0.0280). To better understand how the observed changes in mTOR activation might impact mechanisms related to new protein synthesis, we studied one of its main downstream effectors, the S6 ribosomal protein kinase. We found that S6 phosphorylation levels in serine (S) 240/244 were reduced in the acute phase of the ABA protocol, while no changes were present in its total expression (Figure 6D: −45% vs. CTRL, *t* = 3.629, *p* = 0.0084; Figure 6D: +3% vs. CTRL, *t* = 0.1311, *p* = 0.8989). After the recovery period, both phosphorylation and total expression of S6 were reduced (Figure 6E: −47% vs. CTRL, *t* = 6.975, *p* = 0.0001; Figure 6E: −29% vs. CTRL, *t* = 3.890, *p* = 0.0046). Interestingly, at PND 42 Pearson’s correlations reveal that reduced levels of pTrkB (Figure 5C), pAkt (Figure 5F), and pS6 (Figure 6F) are positively correlated with body weight and food intake (Table 1), while increased levels of pERK2 (Figure 5I) and pmTOR (Figure 6C) are negatively correlated with body weight and food intake (Table 1), indicating that increased levels of mBDNF correspond to reduced food intake and low body weight. The correlation analyses of body weight and the data obtained after the recovery period (TrkB: Figure 5C; Akt: Figure 5F; ERK2: Figure 5I; mTOR: Figure 6C; pS6: Figure 6F) and correlation analysis among body weight and total protein expression levels at both time points (Supplementary Table 6) did not reveal any significant correlation. On the other hand, food intake after recovery negatively correlates with pTrkB, Akt, pERK2, and pS6 (Table 1). Moreover, the Pearson correlation analysis among mBDNF levels measured in the membrane fraction and its downstream targets revealed significant correlations with pTrkB, pAkt, pERK2, and pmTOR in the acute phase of the pathology while positive correlations with pTrkB, TrkB, pAkt, pERK2, pS6, and S6 (Table 3) following a 7-day recovery period.

**Table 3:** Pearson’s product moment coefficients (r), linear regression analyses (R^2^), and p values relative to the correlation between mBDNF protein levels in the membrane fraction with its downstream signaling targets in CTRL and ABA rats at both the achievement of the anorexic phenotype and following a 7-day recovery period.

**mBDNF**	**Acute phase**				**After recovery**			
	**r**	**R^2^**	***p*-value**		**r**	**R^2^**	***p*-value**	
pTrkBtyr706	−0.7682	0.5901	0.0094	**	0.8107	0.6572	0.0044	**
TrkB	0.2804	0.07862	0.4326		0.8505	0.7233	0.0018	**
pAktser473	−0.6586	0.4337	0.0384	*	0.6498	0.4222	0.0420	*
Akt	0.4647	0.2160	0.1760		0.6285	0.3950	0.0516	
pERK2 thr185/tyr187	0.8920	0.7957	0.0005	***	0.8427	0.7102	0.0022	**
ERK2	−0.7290	0.5315	0.0167	*	−0.4398	0.1934	0.2034	
pmTORser2448	0.7533	0.5675	0.0119	*	−0.4372	0.1912	0.2064	
mTOR	0.5958	0.3550	0.0691		0.4684	0.2194	0.1722	
pS6 ser240/244	−0.3331	0.1110	0.3810		0.9070	0.8227	0.0003	***
S6	0.4828	0.2331	0.1575		0.6631	0.4397	0.0366	*

*p < 0.05, **p < 0.01, ***p < 0.001.

**Figure 7:**
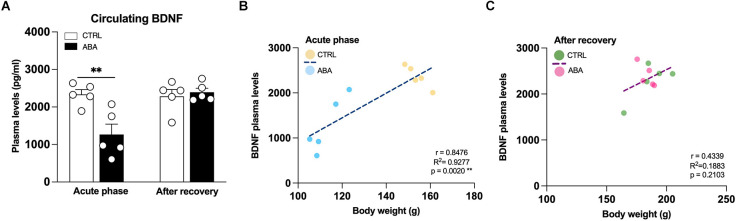
Effects of the ABA protocol on the circulating levels of BDNF of rats exposed to the combination of food restriction and free access to the activity wheel. BDNF plasma levels **(A)** were measured in the acute phase of the disease (PND42) and after a 7-day period of body weight recovery (PND49). Pearson’s product–moment correlation (r) analyses and linear regression analyses (R^2^) between circulating levels of BDNF and body weight of CTRL and ABA rats are measured at PND42 **(B)**, and at PND49 **(C)**. Data are expressed as percentages of control (CTRL) animals. Bar graphs represent the mean ± SEM from five independent determinations for each experimental group. Unpaired Student’s *T* test. *p < 0.05, **p < 0.01, ***p < 0.05. CTRL, control; ABA, activity-based anorexia.

Finally, since clinical studies report reduced circulating levels of BDNF in AN patients (Brandys et al., 2011), with a slight increase or normalization following weight-recovery (Ehrlich et al., 2009), we decided to evaluate BDNF levels in the plasma of ABA rats. Accordingly, circulating levels of BDNF were reduced after the induction of the ABA protocol (Figure 7A: −1,092 pg/ml vs. CTRL, *t* = 3.510, *p* = 0.0080), while no changes are observed after the recovery phase (Figure 7A: +110 pg/ml vs. CTRL, *t* = 0.5126, *p* = 0.6220). Interestingly, Pearson’s correlation analyses show that BDNF plasma levels measured in the acute phase of the disease positively correlated with the measure of body weight at PND42 (Figure 7B), highlighting that low circulating levels of BDNF correspond to a low body weight and* vice versa*. The same analysis performed at PND49 revealed, instead, no significant correlations (Figure 7C).

## Discussion

Our data demonstrate that the induction of the anorexic phenotype in female adolescent rats perturbs the BDNF system in the Amy, an effect that persists even after a period of body weight recovery. These results provide novel neurobiological underpinnings of AN-induced vulnerable phenotype that may contribute to explain, at least in part, the emotional dysregulation in AN and, thus, might be a potential trigger for aberrant behaviors typical of AN patients.

As previously published, the ABA model, i.e., the combination of caloric restriction and physical exercise, induces a food-restriction-evoked hyperactivity thus generating a severe weight loss that, in turn, induces a cycle of self-motivating and compulsive habits promoting hyperactive behaviors despite the severe emaciation (Gutierrez, 2013; Spadini et al., 2021; Zhang and Dulawa, 2021). In fact, the body weight loss of ABA rodents was much greater than the body weight loss of animals exposed to food restriction only, which was also accompanied by an exponential increase in physical activity within 48 h from the beginning of the caloric restriction. The out-of-control behavior that drives rodents to run instead of feeding has been associated with increased anxiety-like behavior both in rodents and in humans, constituting a potential risk factor for more rapid weight loss (Holtkamp et al., 2004; Kaye et al., 2004; Wable et al., 2015; Schwenzer et al., 2022). In addition, higher hyperactivity in the clinic has been linked to a worse prognosis (Scheurink et al., 2010) and to high level of comorbidity with anxiety and depression (Penas-Lledo et al., 2002; Shroff et al., 2006).

In line with human findings, our data suggest that the combination of food restriction and hyperactivity activates the Amy only in the acute phase of the pathology, as shown by an increase in *cfos* mRNA levels, pointing to Amy as an early target of the AN phenotype. Since the elevation of *cfos* expression has been directly associated with natural reward-seeking (Cruz et al., 2013), the amygdalar hyperactivation herein shown might underlie the perpetuation of altered processing of food reward observed in AN patients.

Despite evidence exists that *cfos* is positively associated with *Bdnf* to regulate dendritic development, synapse maturation, synaptic plasticity, and learning and memory (Sommer et al., 1993; Sommer and Fuxe, 1997; Malik et al., 2014), our data indicate that such association appears to fail under the combination of low caloric intake and hyperactivity, as *Bdnf* gene expression is reduced in both the acute phase of the pathology and after a period of body weight recovery. The reduced expression of the neurotrophin, indicating a reduction of the trophic support, may represent a deleterious consequence of the combination of self-starvation and hyperactivity. In addition, the analysis of *Bdnf* mRNA transcripts in the acute phase showed that *Bdnf exon IV*, the most abundant activity-dependent isoform (Pruunsild et al., 2011) localized in the soma, and *exon VI*, known to be targeted to dendrites (Chiaruttini et al., 2008), were reduced, suggesting an overall downregulation of *Bdnf* expression in the Amy. Interestingly, these changes positively correlate with food intake and body weight loss and negatively with wheel running activity indicating that they are involved in sustaining the rapid escalation of maladaptive behaviors in ABA rats. Following the recovery of body weight, the picture is different: despite the persistent reduction of *Bdnf exon IX* in the ABA group, an adaptive mechanism may counteract the reduced trophic support restoring the levels of *exon IV* and* VI*.

Notably, discrepancies exist when examining BDNF transcription and translation. In fact, at the achievement of the anorexic phenotype, reduction in *Bdnf* mRNA levels was accompanied by an upregulation of BDNF protein expression, indicating an AN-induced decoupling among transcriptional and translational mechanisms. The ABA condition increased the translation of mBDNF, measured in the whole homogenate, and its availability at synaptic sites, measured in the membrane fraction, suggesting an accumulation of BDNF in this brain region. Interestingly, a similar effect has been previously shown in the Amy of cocaine-withdrawn rats (Grimm et al., 2003), thus suggesting that the BDNF increase might be a molecular mechanism involved in the onset of compulsive traits observed in the ABA model. Alternatively, the upregulation of mBDNF levels might be a compensatory response to the primary lack of BDNF transcription. After 7 days of body weight recovery, despite the reduced *Bdnf* gene expression still persisting, no changes in the mBDNF protein were observed in the whole homogenate whereas reduced levels of mBDNF were measured at synaptic sites of ABA rodents. Since the total amount of mBDNF in the cell was comparable to controls, the reduced mBDNF levels in the membrane might be due to a more rapid turnover or increased protein degradation, suggesting that, despite rats undergoing a recovery period, the combination of food restriction and excessive physical exercise persistently reduces the pool of BDNF ready to be released. These data suggest that, after recovery, ABA animals lack a trophic supply, an effect that might compromise synaptic plasticity in this brain area.

**Figure 8:**
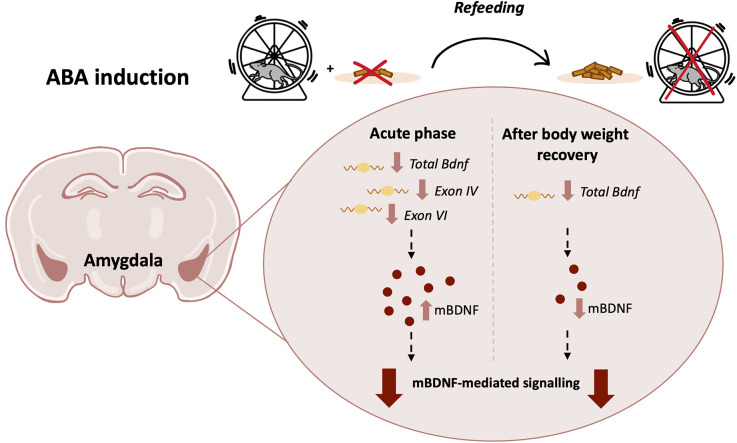
Graphical representation of the ABA protocol with a summary of the main effects on the BDNF system in the Amygdala of adolescent female rats in the acute phase of the anorexic phenotype and after a period of body weight recovery (ABA, activity-based anorexia; BDNF, brain-derived neurotrophic factor).

We then extended our investigation by analyzing the BDNF-mediated downstream signaling pathways. In fact, in the acute phase of the pathology, we found reduced phosphorylation of TrkB, an index of activation upon neurotrophin release (Saarelainen et al., 2003), reduced phosphorylation of Akt and S6 in ABA rats, a canonical pathway known to be activated by BDNF and promote protein synthesis (Gong et al., 2006; Costa-Mattioli and Monteggia, 2013). This finding points to TrkB-Akt-S6 cascade as a primary target of anorexic phenotype in the Amy. Notably, the combination of food restriction and exercise significantly increased the phosphorylation of ERK2 and mTOR. Since hyperphosphorylation of both ERK and mTOR has been correlated to pro-apoptotic processes, mitochondrial damage, depression, and early life stress (Duman et al., 2012; Bordi et al., 2019; Martin-Sanchez et al., 2022), we can hypothesize that the induction of the anorexic phenotype might drive similar effects, which need further detailed investigation. Moreover, increased activation of ERK2 might be interpreted as a marker of reward and seeking behaviors since it is activated in several brain regions, including the Amy, following cocaine exposure (Valjent et al., 2000; Lu et al., 2006; Giannotti et al., 2014). Thus, the ABA-induced activation of the ERK pathway might contribute to compulsive behaviors observed in ABA rats. Following the recovery period, akin to reduced BDNF levels, ABA-recovered rodents show an overall reduction of the targets downstream BDNF. In fact, pTrkB reduction, in turn, resulted in a decrease of both ERK2- and Akt-dependent pathways that subsequently decreased S6 phosphorylation. These results suggest that the combination of body weight loss and hyperactivity permanently alters the physiological regulation of the translational machinery, an effect that persists even after a 7-day period of recovery. Taken together, our data highlight the widespread impact on neuroplasticity exerted by the ABA condition: the overall downregulation of the amygdalar BDNF machinery may reflect reduced synaptic transmission thus leading to long-lasting instability (Figure 8). Moreover, reduced levels of BDNF and its mediated signaling pathway suggest an impairment in Amy-dependent learning, such as appetitive and fear learning. Since BDNF contributes to reducing inhibition and increasing excitability necessary for fear memory formation by inducing a rapid internalization of GABA(A)R α1 subunits (Mou et al., 2011, 2013), the ABA-induced BDNF deficit may alter the inhibition-excitation balance in the Amy, thus compromising memory formation (Meis et al., 2020).

Interestingly, BDNF is abundantly present at the peripheral level in the blood flow (Fujimura et al., 2002). Despite in most cases peripheral and neuronal BDNF levels appear to correlate (Klein et al., 2011), in our experimental condition we found a discrepancy between BDNF plasma levels and its amount in the Amy. In fact, in the acute phase, the increased amygdalar levels were paralleled by reduced plasmatic levels whereas, following recovery, no changes were observed both in the amygdalar homogenate and in the plasma of ABA rats. Despite the herein shown dichotomy, our data are in line with clinical studies showing decreased BDNF serum levels in the acute stage of the pathology (Brandys et al., 2011; Monteleone and Maj, 2013). Due to BDNF-mediated modulation of anorexigenic and orexigenic pathways in appetite and satiety processes (Pelleymounter et al., 1995; Rosas-Vargas et al., 2011), the decreased circulating levels of BDNF may represent a maladaptive phenomenon to dysregulate food intake even in a condition of starvation. Moreover, low BDNF circulating levels have also been reported in other chronic mental disorders, including obsessive-compulsive disorder, major depression, bipolar disorder, and schizophrenia (Bocchio-Chiavetto et al., 2010; Fontenelle et al., 2012; Brand et al., 2015; Fernandes et al., 2015), reasonably pointing to BDNF as a potential biomarker of increased vulnerability also in AN. Regarding the modulation of BDNF after weight recovery, different investigations found a normalization of serum BDNF in weight-recovered AN patients (Ehrlich et al., 2009; Zwipp et al., 2014; Tyszkiewicz-Nwafor et al., 2019) or a rise to supranormal levels that are significantly higher than those of healthy controls (Borsdorf et al., 2021). Even though the human condition is less clear, body weight recovery in ABA rats restored circulating BDNF levels, suggesting that, in rats, peripheral mechanisms mediating BDNF homeostasis are still able to recover.

Regardless of the evidence that BDNF Val66Met polymorphism does not increase susceptibility in the onset of AN symptomatology, such as feeding and exercise, in the ABA rat model (Chen et al., 2017; Pietrucci et al., 2022), the significant correlation among BDNF levels and body weight, food intake and distance traveled on the wheel in the acute phase of the pathology provide evidence for a role of BDNF in the regulation of food intake and energy homeostasis at both central and peripheral level. Interestingly, even though body weight recovery was paralleled by restored circulating BDNF levels, a dysregulation at the central level persists, suggesting BDNF as an AN-induced molecular scar of maladaptive plasticity. Moreover, our correlation analysis among the ABA-induced behavioral outcomes and the BDNF system at multiple levels revealed that the molecular dysregulation of the BDNF system observed at both the achievement of the anorexic phenotype and following a 7-day recovery period further corroborate the AN-induced weakening of trophic support in the Amy as a signature of ABA-induced vulnerability.

Interestingly, given the role of BDNF in psychiatric disorders (Wang et al., 2022), our findings might pave the way for a better comprehension of the molecular mechanisms underlying comorbid psychiatric disorders including mood disorder, social phobia, obsessive-compulsive disorder and substance use disorder (Jagielska and Kacperska, 2017) that persist beyond recovery from AN (Braun et al., 1994; Herzog et al., 1996; Marucci et al., 2018) and that might be linked to poor outcome in patients. In particular, given the role of the Amy in stress responsivity and emotionality and since during adolescence, when the onset of AN peaks (Micali et al., 2013), this brain area undergoes refinement processes (Lenroot and Giedd, 2006), interfering with its maturational trajectory might alter the development of adaptive emotion regulation strategies (Ahmed et al., 2015), typical of AN patients. In fact, similarly to stressful life events and drug exposure (Heim and Nemeroff, 2001; Salmanzadeh et al., 2020; Caffino et al., 2022), the experience of ABA during adolescence, but not in adulthood, induces long-term anxiety-like behavior measured as reduced entries in the open arms of an elevated plus maze and reduced time spent in the center of an open field arena (Kinzig and Hargrave, 2010) *via* dysregulation of brain homeostasis. Other risk factors for ABA-induced vulnerability are represented by psychiatric comorbidities, such as anhedonia, a trait reported also in patients with AN (Milton et al., 2018), impairment in cognitive functions such as learning and memory (Boersma et al., 2016; Paulukat et al., 2016; Mottarlini et al., 2022) and cognitive flexibility (Allen et al., 2017; Milton et al., 2018) and in reward-related behaviors *via* alteration in the midbrain reward circuitry (Lett et al., 2000; Foldi et al., 2017). Similar to individuals who have recovered from AN and continue to show higher levels of anxiety (Kaye et al., 2004), ABA induction increases anxiety-like behavior also after a recovery period (Chen et al., 2017, 2018), an effect that might be driven by the herein shown persistent changes in the BDNF system in the Amy.

We are aware that our study is limited by the lack of direct evidence of BDNF involvement in the induction of anxiety-like behavior in ABA rats; however, our data add complexity as well as specificity to the involvement of the BDNF system in the maintenance of the anorexic phenotype and in the increased vulnerability of AN patients. Moreover, since AN is a chronic disease that might last several years in humans and chronic ABA models (Frintrop et al., 2018; Schalla and Stengel, 2019), to mimic more closely the impact of AN on many somatic aspects that are not manifested in the acute model used in the present study, have been established, we cannot rule out that the herein shown changes in the BDNF system might contribute to the long-term vicious cycle of self-starvation/body weight loss and hyperactivity in chronic AN. Further studies taking advantage of the long-term ABA protocol should implement this gap of knowledge. Thus, the comprehension of the molecular mechanisms underlying the interplay of AN and anxiety disorder might provide important features in the pathophysiology of AN.

Since recent data reveal an increase of AN cases even in adolescent males (Gorrell and Murray, 2019) and only a few articles on humans and the ABA model focused on the difference between male and female subjects (Perez-Leighton et al., 2014; Timko et al., 2019; Morgan et al., 2022), researchers largely ignore potentially differences in the development and maintenance of AN in males and females. Nevertheless, a more detailed understanding of neurobiological factors involved in sex differences and in the different phases of AN pathology needs to be further addressed in future research to develop more targeted treatment strategies.

## Data Availability Statement

The original contributions presented in the study are included in the article/Supplementary material, further inquiries can be directed to the corresponding author.

## Ethics Statement

The animal study was reviewed and approved by Organismo Preposto al Benessere degli Animali—OPBA Università degli Studi di Milano—Ministero della Salute.

## Author Contributions

FM, FF, and LC: conceptualization. FM, BR, and GT: methodology. FM and BR: software. FM, BR, GT, and LC: investigation. FM and LC: writing—original draft preparation. FM, BR, GT, FF, and LC: writing—review and editing. FF and LC: supervision and funding acquisition. All authors contributed to the article and approved the submitted version.
