# Supplementary material for: Long-lasting BDNF signaling alterations in the amygdala of adolescent female rats exposed to the activity-based anorexia model
Source: Front Behav Neurosci. 2022 Dec 8;16:1087075. doi: 10.3389/fnbeh.2022.1087075 (PMC9772010; doi:10.3389/fnbeh.2022.1087075)
Supplement: Supplementary file 1 [file Data_Sheet_1.docx]

Supplementary Material

# Measure of food intake during the ABA protocol

## Food intake

**Supplementary Figure 1.** Average daily food intake measured in grams of food in control (CTRL), food-restricted (FR), exercise (EXE) and ABA rats. Results are presented as the mean ± SEM of five rats per group. Two-way ANOVA, followed by Bonferroni’s multiple comparisons test. °° p < 0,01, °°° p < 0.001 ABA vs CTRL; $$$ p < 0.001 ABA vs EXE; & p < 0.05, && p < 0.01 FR vs ABA; ### p < 0.001 FR and ABA vs CTRL and EXE.

CTRL (control); FR (food-restricted); EXE (exercise); ABA (activity-based anorexia).

# Western blot images

3= CTRL-recovery

2= ABA-acute

1= CTRL-acute

4= ABA-recovery

50 -

75 -

37 -

mBDNF (14 kDa)

15 -

10 -

β-actin (43 kDa)

25 -


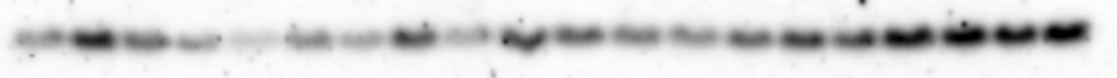

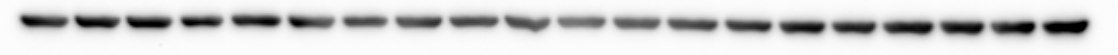


1

4

1

4

2

3

2

3

1

1

4

2

3

2

3

4

1

4

2

3

1

4

1

4

2

3

2

3

1

1

4

2

3

2

3

4

1

4

2

3

**Supplementary Figure 2.** Cropped immunoblot related to the expression levels of mBDNF and β-actin measured in the homogenate of Amy of CTRL and ABA animals in the acute (P42) and after a 7-days recovery period (P49), presented in Figure 4.

3= CTRL-recovery

2= ABA-acute

1= CTRL-acute

4= ABA-recovery

50 -

100 -

75 -

150 -

150 -

37 -

mBDNF (14 kDa)

15 -

10 -

β-actin (43 kDa)

Akt (60 kDa)

pAkts473 (60 kDa)

pERK2 (42 kDa)

ERK2 (42 kDa)

pTrkBy706 (145 kDa)

TrkB (145 kDa)

75 -

50 -

50 -

1

4

1

4

2

3

2

3

1

1

4

2

3

2

3

4

1

4

2

3

1

4

1

4

2

3

2

3

1

1

4

2

3

2

3

4

1

4

2

3

25 -


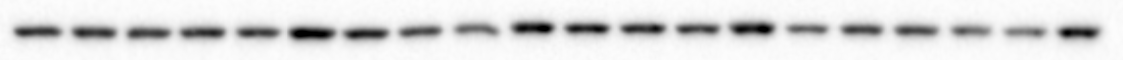

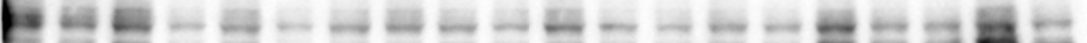

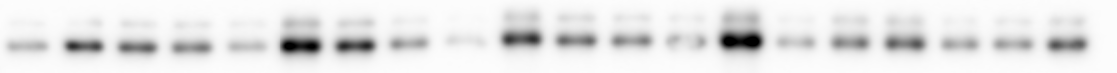

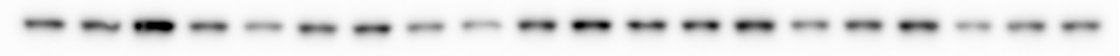

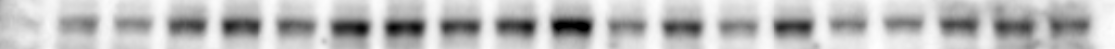


1

4

1

4

2

3

2

3

1

1

4

2

3

2

3

4

1

4

2

3

1

4

1

4

2

3

2

3

1

1

4

2

3

2

3

4

1

4

2

3

1

4

1

4

2

3

2

3

1

1

4

2

3

2

3

4

1

4

2

3

1

4

1

4

2

3

2

3

1

1

4

2

3

2

3

4

1

4

2

3

1

4

1

4

2

3

2

3

1

1

4

2

3

2

3

4

1

4

2

3

1

4

1

4

2

3

2

3

1

1

4

2

3

2

3

4

1

4

2

3


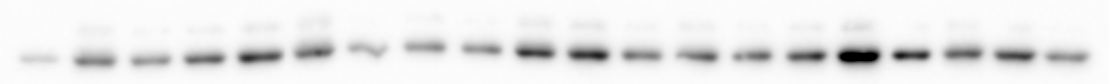

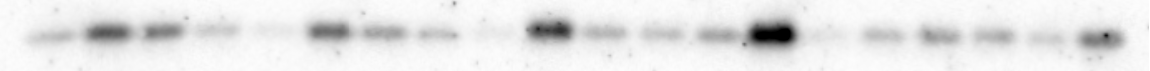


250 -


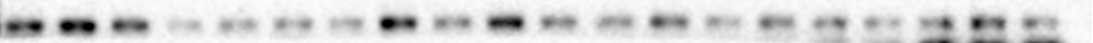


**Supplementary Figure 3.** Cropped immunoblot related to the expression levels of pTrkBy706, TrkB, pAkts473, Akt, pERK2, ERK2, mBDNF and β-actin measured in the crude membrane fraction of Amy of CTRL and ABA animals in the acute (P42) and after a 7-days recovery period (P49), presented in Figure 4 and 5.

50 -

100 -

250 -

75 -

150 -

37 -

37 -

β-actin (43 kDa)

pS6s244/240 (32 kDa)

S6 (32 kDa)

25 -


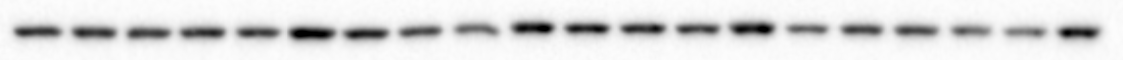

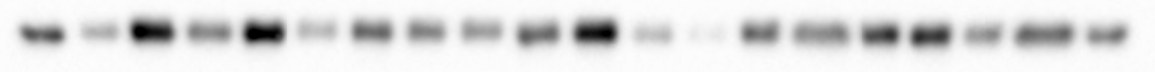

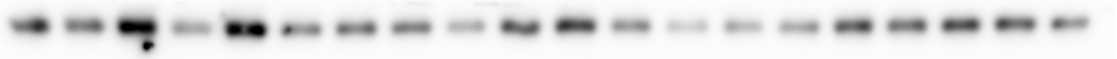


pmTORs2448 (289 kDa)

mTOR (289 kDa)

250 -


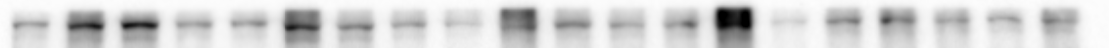

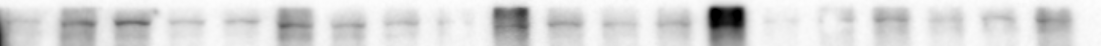


1

4

1

4

2

3

2

3

1

1

4

2

3

2

3

4

1

4

2

3

1

4

1

4

2

3

2

3

1

1

4

2

3

2

3

4

1

4

2

3

1

4

1

4

2

3

2

3

1

1

4

2

3

2

3

4

1

4

2

3

1

4

1

4

2

3

2

3

1

1

4

2

3

2

3

4

1

4

2

3

1

4

1

4

2

3

2

3

1

1

4

2

3

2

3

4

1

4

2

3

3= CTRL-recovery

2= ABA-acute

1= CTRL-acute

4= ABA-recovery

50 -

100 -

250 -

75 -

150 -

37 -

37 -

β-actin (43 kDa)

pS6s244/240 (32 kDa)

S6 (32 kDa)

25 -


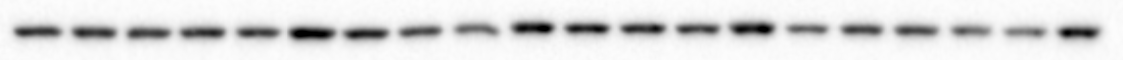

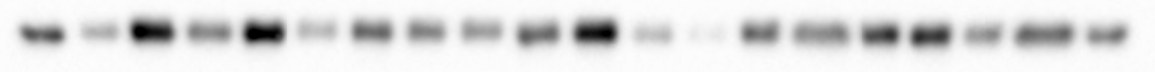

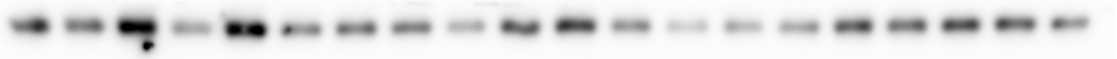


pmTORs2448 (289 kDa)

mTOR (289 kDa)

250 -


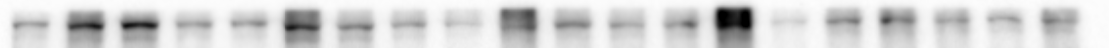

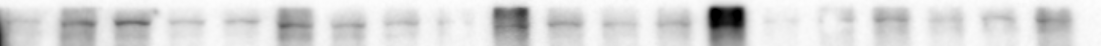


1

4

1

4

2

3

2

3

1

1

4

2

3

2

3

4

1

4

2

3

1

4

1

4

2

3

2

3

1

1

4

2

3

2

3

4

1

4

2

3

1

4

1

4

2

3

2

3

1

1

4

2

3

2

3

4

1

4

2

3

1

4

1

4

2

3

2

3

1

1

4

2

3

2

3

4

1

4

2

3

1

4

1

4

2

3

2

3

1

1

4

2

3

2

3

4

1

4

2

3

**Supplementary Figure 4.** Cropped immunoblot related to the expression levels of pmTOR s2448, mTOR, pS6 s244/240, S6, mBDNF, β-actin measured in the crude membrane fraction of Amy of CTRL and ABA animals in the acute (P42) and after a 7-days recovery period (P49), presented in Figure 6.

# Statistical analyses

## Food intake, body weight and distance travel measurements

| **Food intake (g)** | | | Bonferroni's multiple comparisons test | |
| --- | --- | --- | --- | --- |
| **Post-natal day (PND)** | **Groups** | | **Mean difference (%)** | **Adjusted p value** |
| 39 | | CTRL vs. FR | 17,68 | <0,0001 |
|  | | CTRL vs. ABA | 15,52 | <0,0001 |
|  | | FR vs. EXE | -18,68 | <0,0001 |
|  | | EXE vs. ABA | 16,52 | <0,0001 |
| 40 | | CTRL vs. FR | 14,52 | <0,0001 |
|  | | CTRL vs. ABA | 14,40 | <0,0001 |
|  | | FR vs. EXE | -17,11 | <0,0001 |
|  | | EXE vs. ABA | 16,98 | <0,0001 |
| 41 | | CTRL vs. FR | 13,58 | <0,0001 |
|  | | CTRL vs. ABA | 13,65 | <0,0001 |
|  | | FR vs. EXE | -15,27 | <0,0001 |
|  | | EXE vs. ABA | 15,34 | <0,0001 |
| 42 | | CTRL vs. FR | 12,09 | <0,0001 |
|  | | CTRL vs. ABA | 12,71 | <0,0001 |
|  | | FR vs. EXE | -15,75 | <0,0001 |
|  | | EXE vs. ABA | 16,38 | <0,0001 |
| 46 | | CTRL vs. ABA | -5,866 | 0,0053 |
| 47 | | CTRL vs. ABA | -6,440 | 0,0010 |
|  | | FR vs. ABA | -5,558 | 0,0123 |
| 49 | | CTRL vs. ABA | -7,022 | 0,0002 |
|  | | FR vs. ABA | -5,756 | 0,0072 |
|  | | EXE vs. ABA | -6,698 | 0,0005 |

**Supplementary Table 1.** Detailed statistical values of data presented in supplementary figure 1. Mean differences and adjusted p values relative to Bonferroni’s multiple comparisons test on the analysis of food intake measured in grams (g).

| **Body weight** | | Bonferroni's multiple comparisons test | |
| --- | --- | --- | --- |
| **Post-natal day (PND)** | **Groups** | **Mean difference (%)** | **Adjusted p value** |
| 39 | CTRL vs. ABA | 25,14 | 0,0007 |
| 40 | CTRL vs. FR | 26,18 | 0,0003 |
|  | CTRL vs. ABA | 41,68 | <0,0001 |
|  | EXE vs. ABA | 31,04 | <0,0001 |
| 41 | CTRL vs. FR | 36,24 | <0,0001 |
|  | CTRL vs. ABA | 58,12 | <0,0001 |
|  | FR vs. EXE | -23,78 | 0,0021 |
|  | FR vs. ABA | 21,88 | 0,0090 |
|  | EXE vs. ABA | 45,66 | <0,0001 |
| 42 | CTRL vs. FR | 43,06 | <0,0001 |
|  | CTRL vs. ABA | 64,82 | <0,0001 |
|  | FR vs. EXE | -32,00 | <0,0001 |
|  | FR vs. ABA | 21,76 | 0,0099 |
|  | EXE vs. ABA | 53,76 | <0,0001 |
| 43 | CTRL vs. FR | 34,52 | <0,0001 |
|  | CTRL vs. ABA | 57,26 | <0,0001 |
|  | FR vs. EXE | -20,76 | 0,0204 |
|  | FR vs. ABA | 22,74 | 0,0047 |
|  | EXE vs. ABA | 43,50 | <0,0001 |
| 44 | CTRL vs. FR | 28,14 | <0,0001 |
|  | CTRL vs. ABA | 47,24 | <0,0001 |
|  | EXE vs. ABA | 35,82 | <0,0001 |
|  | CTRL vs. FR | 27,80 | <0,0001 |
|  | CTRL vs. ABA | 42,66 | <0,0001 |
|  | EXE vs. ABA | 29,04 | <0,0001 |
| 46 | CTRL vs. FR | 23,54 | 0,0026 |
|  | CTRL vs. ABA | 37,22 | <0,0001 |
|  | EXE vs. ABA | 25,66 | 0,0005 |
| 47 | CTRL vs. FR | 24,62 | 0,0011 |
|  | CTRL vs. ABA | 36,44 | <0,0001 |
|  | EXE vs. ABA | 21,92 | 0,0088 |
| 48 | CTRL vs. FR | 22,90 | 0,0042 |
|  | CTRL vs. ABA | 33,42 | <0,0001 |
|  | EXE vs. ABA | 18,64 | 0,0858 |
| 49 | CTRL vs. FR | 22,86 | 0,0043 |
|  | CTRL vs. ABA | 26,64 | 0,0002 |

**Supplementary Table 2.** Detailed statistical values of data presented in figure 1B. Mean differences and adjusted p values relative to Bonferroni’s multiple comparisons test the analysis of body weight.

| **Bonferroni's multiple comparisons test** | |  | |  |
| --- | --- | --- | --- | --- |
| **Post-natal day (PND)** | **Predicted (LS) mean diff,** | | **Adjusted p Value** | |
|  |  | |  | |
| EXE vs ABA 40 | -8779 | | 0,0081 | |
| 41 | -15379 | | <0,0001 | |
| 42 | -17765 | | <0,0001 | |

**Supplementary Table 3.** Detailed statistical values of data presented in figure 1D. Mean differences and adjusted p values relative to Bonferroni’s multiple comparisons test related to the distance travelled by exercise animals (EXE) vs ABA rats.

| **Three-way ANOVA** | | **F value** | **P value** | |
| --- | --- | --- | --- | --- |
| ***cfos*** | time | F (1, 32) = 43,60 | P<0,0001 | **** |
|  | exercise | F (1, 32) = 10,50 | P=0,0028 | ** |
|  | food restriction | F (1, 32) = 0,7719 | P=0,3862 |  |
|  | time x exercise | F (1, 32) = 0,03222 | P=0,8587 |  |
|  | time x food restriction | F (1, 32) = 12,95 | P=0,0011 | ** |
|  | exercise x food restriction | F (1, 32) = 5,357 | P=0,0272 | * |
|  | time x exercise x food restriction | F (1, 32) = 0,09718 | P=0,7573 |  |
| ***Bdnf exon IX*** | time | F (1, 32) = 0,06850 | P=0,7952 |  |
|  | exercise | F (1, 32) = 4,242 | P=0,0476 | * |
|  | food restriction | F (1, 32) = 14,36 | P=0,0006 | *** |
|  | time x exercise | F (1, 32) = 0,06850 | P=0,7952 |  |
|  | time x food restriction | F (1, 32) = 0,06850 | P=0,7952 |  |
|  | exercise x food restriction | F (1, 32) = 50,58 | P<0,0001 | **** |
|  | time x exercise x food restriction | F (1, 32) = 0,06850 | P=0,7952 |  |
| ***Bdnf exon IV*** | time | F (1, 32) = 6,940 | P=0,0129 | * |
|  | exercise | F (1, 32) = 6,362 | P=0,0168 | * |
|  | food restriction | F (1, 32) = 8,826 | P=0,0056 | ** |
|  | time x exercise | F (1, 32) = 0,4276 | P=0,5178 |  |
|  | time x food restriction | F (1, 32) = 2,891 | P=0,0988 |  |
|  | exercise x food restriction | F (1, 32) = 16,14 | P=0,0003 | *** |
|  | time x exercise x food restriction | F (1, 32) = 5,282 | P=0,0282 | * |
| ***Bdnf exon VI*** | time | F (1, 32) = 0,8953 | P=0,3511 |  |
|  | exercise | F (1, 32) = 4,935 | P=0,0335 | * |
|  | food restriction | F (1, 32) = 7,150 | P=0,0117 | * |
|  | time x exercise | F (1, 32) = 9,775 | P=0,0038 | ** |
|  | time x food restriction | F (1, 32) = 5,120 | P=0,0306 | * |
|  | exercise x food restriction | F (1, 32) = 20,48 | P<0,0001 | **** |
|  | time x exercise x food restriction | F (1, 32) = 2,708 | P=0,1096 |  |

**Supplementary Table 4.** Detailed F values, degrees of freedom and p values relative to three-way ANOVA analyses of genes expression measured in the Amygdala of CTRL, FR, EXE and ABA rats and presented in figure 2 and 3.

|  |  | **Acute phase** | | | **After recovery** | | |
| --- | --- | --- | --- | --- | --- | --- | --- |
| Two-way ANOVA | | **F value** | **P value** | | **F value** | **P value** | |
| ***cfos*** | Interaction | F (1, 16) = 4,777 | P=0,0441 | * | F (1, 16) = 1,569 | P=0,2283 |  |
|  | Exercise | F (1, 16) = 8,099 | P=0,0117 | * | F (1, 16) = 3,665 | P=0,0736 |  |
|  | Food restriction | F (1, 16) = 13,89 | P=0,0018 | ** | F (1, 16) = 2,896 | P=0,1082 |  |
| ***Bdnf exon IX*** | Interaction | F (1, 16) = 31,56 | P<0,0001 | **** | F (1, 16) = 9,629 | P=0,0068 | ** |
|  | Exercise | F (1, 16) = 2,174 | P=0,1597 |  | F (1, 16) = 6,147 | P=0,0247 | * |
|  | Food restriction | F (1, 16) = 8,370 | P=0,0106 | * | F (1, 16) = 5,254 | P=0,0358 | * |
| ***Bdnf exon VI*** | Interaction | F (1, 16) = 25,98 | P=0,0001 | *** | F (1, 16) = 3,272 | P=0,0893 |  |
|  | Exercise | F (1, 16) = 19,52 | P=0,0004 | *** | F (1, 16) = 0,3232 | P=0,5776 |  |
|  | Food restriction | F (1, 16) = 16,63 | P=0,0009 | *** | F (1, 16) = 0,06678 | P=0,7994 |  |

**Supplementary Table 5.** Detailed F values, degrees of freedom and p values relative to two-way ANOVA analyses of genes expression measured in the Amygdala of CTRL, FR, EXE and ABA rats at the achievement of the acute phase and after a seven-day recovery period and presented in figure 2 and 3.

|  | **Body weight (g)** | acute phase | | | | after recovery | | |
| --- | --- | --- | --- | --- | --- | --- | --- | --- |
|  | | **r** | **R^2^** | **p value** | | **r** | **R^2^** | **p value** |
|  | TrkB | -0,03751 | 0,001407 | 0,9181 |  | 0,3717 | 0,1381 | 0,2903 |
|  | Akt | -0,3427 | 0,1174 | 0,3324 |  | 0,1621 | 0,02626 | 0,6547 |
|  | ERK2 | 0,8422 | 0,7093 | 0,0022 | ** | 0,5341 | 0,2852 | 0,1118 |
|  | mTOR | -0,5583 | 0,3117 | 0,0935 |  | 0,2509 | 0,06297 | 0,4844 |
|  | S6 | -0,06873 | 0,004723 | 0,8504 |  | 0,4955 | 0,2455 | 0,1453 |

**Supplementary Table 6.** Pearson’s product moment coefficients (r), linear regression analyses (R^2^) and p values relative to the correlation among body weight measured in grams (g) and total protein expression levels in CTRL and ABA rats at both the achievement of the anorexic phenotype and following a seven-day recovery period.
